# 2,2′-Dimethoxy-4,4′-[*rel*-(2*R*,3*S*)-2,3-di­methylbutane-1,4-diyl]diphenol

**DOI:** 10.1107/S1600536809017292

**Published:** 2009-05-14

**Authors:** Carmen L. Salinas-Salazar, María del Rayo Camacho-Corona, Sylvain Bernès, Noemi Waksman de Torres

**Affiliations:** a Laboratorio de Química de Productos Naturales, DEP Facultad de Ciencias Químicas, UANL, Monterrey, N.L., Mexico; b Laboratorio de Difracción de rayos X en Monocristales, DEP Facultad de Ciencias Químicas, UANL, Monterrey, N.L., Mexico; c Departamento de Química Analítica, Facultad de Medicina, UANL, Monterrey, N.L., Mexico

## Abstract

The title mol­ecule, C_20_H_26_O_4_, commonly known as *meso*-dihydro­guaiaretic acid, is a naturally occurring lignan extracted from *Larrea tridentata* and other plants. The mol­ecule has a noncrystallographic inversion center situated at the midpoint of the central C—C bond, generating the *meso* stereoisomer. The central C—C—C—C alkyl chain displays an all-*trans* conformation, allowing an almost parallel arrangement of the benzene rings, which make a dihedral angle of 5.0 (3)°. Both hydr­oxy groups form weak O—H⋯O—H chains of hydrogen bonds along [100]. The resulting supra­molecular structure is an undulating plane parallel to (010).

## Related literature

For the extraction of the title mol­ecule from *Larrea tridentata*, see: Waller & Gisvold (1945 ▶). For previous phytochemical characterizations, see: Gnabre *et al.* (1995 ▶); Konno *et al.* (1990 ▶); Tyler & Foster (1999 ▶). For the activity of this plant against *Mycobacterium tuberculosis*, see: Camacho-Corona *et al.* (2008 ▶).
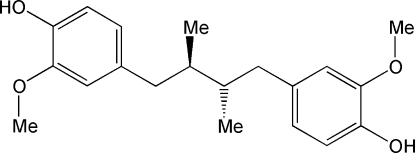


## Experimental

### 

#### Crystal data

C_20_H_26_O_4_
*M* *_r_* = 330.41
Orthorhombic, 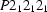

*a* = 5.1355 (8) Å
*b* = 12.024 (2) Å
*c* = 30.158 (5) Å
*V* = 1862.2 (5) Å^3^
*Z* = 4
Mo *K*α radiation
μ = 0.08 mm^−1^
*T* = 298 K
0.50 × 0.40 × 0.18 mm

#### Data collection

Siemens P4 diffractometer
Absorption correction: none
3966 measured reflections
1937 independent reflections
1196 reflections with *I* > 2σ(*I*)
*R*_int_ = 0.159
2 standard reflections every 98 reflections intensity decay: 1%

#### Refinement

*R*[*F* ^2^ > 2σ(*F* ^2^)] = 0.054
*wR*(*F* ^2^) = 0.139
*S* = 1.00
1937 reflections
227 parameters
2 restraints
H atoms treated by a mixture of independent and constrained refinement
Δρ_max_ = 0.18 e Å^−3^
Δρ_min_ = −0.17 e Å^−3^

### 

Data collection: *XSCANS* (Siemens, 1996 ▶); cell refinement: *XSCANS*; data reduction: *XSCANS*; program(s) used to solve structure: *SHELXS97* (Sheldrick, 2008 ▶); program(s) used to refine structure: *SHELXL97* (Sheldrick, 2008 ▶); molecular graphics: *SHELXTL* (Sheldrick, 2008 ▶) and *Mercury* (Macrae *et al.*, 2006 ▶); software used to prepare material for publication: *SHELXL97*.

## Supplementary Material

Crystal structure: contains datablocks I, global. DOI: 10.1107/S1600536809017292/is2391sup1.cif

Structure factors: contains datablocks I. DOI: 10.1107/S1600536809017292/is2391Isup2.hkl

Additional supplementary materials:  crystallographic information; 3D view; checkCIF report

## Figures and Tables

**Table 1:** Hydrogen-bond geometry (Å, °)

*D*—H⋯*A*	*D*—H	H⋯*A*	*D*⋯*A*	*D*—H⋯*A*
O2—H2⋯O14^i^	0.84 (2)	2.15 (3)	2.908 (6)	149 (5)
O14—H14⋯O2^ii^	0.86 (2)	2.35 (4)	3.030 (5)	137 (5)

Symmetry codes: (i) 
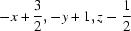
; (ii) 
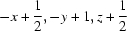
.

**Fig. 1.**
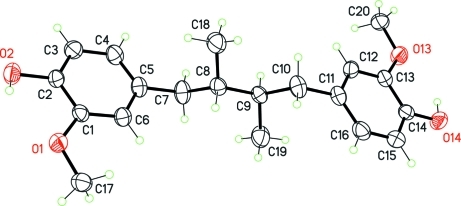
The title compound, with displacement ellipsoids at the 30% probability level.

**Fig. 2.**
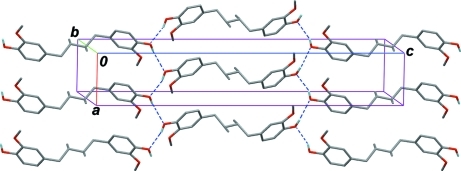
A part of the crystal structure of the title compound. For H atoms, only hydroxy H atoms have been retained, which are engaged in hydrogen bonding (dashed bonds).

## supplementary crystallographic information

### Comment

*Larrea tridentata*, also known as gobernadora, hediondilla, greasewood, chaparral or creosote bush, is a shrubby plant belonging to the family of Zygophyllaceae, which grows in some areas of the desert southwest in the United States of America and Northern Mexico. Tuberculosis, cancer, menstrual pains, and diabetes treatment are among the indications listed for chaparral (Tyler & Foster, 1999). For instance, *L. tridentata* has been shown to be active against *Mycobacterium tuberculosis*, with a minimum inhibitory concentration of 200 µg/ml (Camacho-Corona *et al.*, 2008). We are currently working on the full characterization of the main active compounds found in the chloroform extract of that plant.

Previous phytochemical studies carried out on *L. tridentata* showed that it contains a series of lignans (Konno *et al.*, 1990; Gnabre *et al.*, 1995), one of which being the title molecule. This molecule, commonly called *meso*-dihydroguaiaretic acid, crystallizes in the space group *P*2_1_2_1_2_1_, with the molecule placed on a non-crystallographic inversion center (Fig. 1). As a consequence, the relative stereochemistry for chiral C atoms is (*R*,*S*). The central aliphatic chain is stabilized in an all-*trans* conformation, and peripheral benzene rings are almost parallel, making a dihedral angle of 5.0 (3)°.

The crystal structure features weak O—H···O hydrogen bonds involving all hydroxy groups. Infinite chains are formed along the short axis [100], with OH functionalities serving both as donor and acceptor groups. As a result, a two-dimensional supramolecular framework is formed, parallel to plane (010) in the crystal (Fig. 2).

### Experimental

Aerial parts of *L. tridentata* were collected in April 2006, at Galeana (Nuevo León, Mexico) and identified by Biologist Marcela González Álvarez. A voucher specimen (024772) is available in the botanic department of the Biology Faculty (UNL, Monterrey, Mexico). After grinding, the dry material (500 g) was placed in an Erlenmeyer vessel filled with hexane (1 l) and left at 298 K for 24 h. The preparation was then filtered and the resulting vegetal material soaked with chloroform for 72 h. The chloroform extract was then filtered and concentrated *in vacuo*, affording 89 g of extracts. The chloroform extract (80 g) was chromatographed on silica-gel (1600 g) using mixtures of chloroform/ethanol as eluent, giving 13 fractions. The third fraction, eluted with pure CHCl_3_, afforded colorless crystals, which were separated by filtration. After recrystallization from hexane/ethyl acetate (80:20), pure *meso*-dihydroguaiaretic acid was obtained (730 mg, m.p. 360 K). Spectroscopic data are consistent with the X-ray structure (see archived CIF). The full characterization by NMR also allowed to confirm that this lignan was early isolated by Waller & Gisvold (1945) from the same plant.

### Refinement

As no significant anomalous scattering effects are present in the crystal, measured Friedel pairs (1325) were merged for refinement. Hydroxyl H atoms, H2 and H14, were found in a difference map and refined freely, although O—H bond lengths were restrained to 0.85 (2) Å. Other H atoms were placed in idealized positions and refined as riding to their parent C atom, with bond lengths fixed to 0.93 (aromatic CH), 0.97 (methylene CH_2_) or 0.96 Å (methyl CH_3_). Methyl groups were considered as rigid groups free to rotate about their C—C bonds. Isotropic displacement parameters for H atoms were calculated as *U*_iso_(H) = 1.5*U*_eq_(carrier atom) for methyl and hydroxyl groups and *U*_iso_(H) = 1.2*U*_eq_(carrier atom) otherwise.

### Crystal data

**Table:**

C_20_H_26_O_4_	*D*_x_ = 1.179 Mg m^−^^3^
*M**_r_* = 330.41	Melting point: 360 K
Orthorhombic, *P*2_1_2_1_2_1_	Mo *K*α radiation, λ = 0.71073 Å
Hall symbol: P 2ac 2ab	Cell parameters from 54 reflections
*a* = 5.1355 (8) Å	θ = 4.4–11.0°
*b* = 12.024 (2) Å	µ = 0.08 mm^−^^1^
*c* = 30.158 (5) Å	*T* = 298 K
*V* = 1862.2 (5) Å^3^	Plate, colourless
*Z* = 4	0.50 × 0.40 × 0.18 mm
*F*(000) = 712	

### Data collection

**Table:**

Siemens P4 diffractometer	*R*_int_ = 0.159
Radiation source: fine-focus sealed tube	θ_max_ = 25.0°, θ_min_ = 1.8°
graphite	*h* = −6→6
ω scans	*k* = −14→1
3966 measured reflections	*l* = −35→1
1937 independent reflections	2 standard reflections every 98 reflections
1196 reflections with *I* > 2σ(*I*)	intensity decay: 1%

### Refinement

**Table:**

Refinement on *F*^2^	Primary atom site location: structure-invariant direct methods
Least-squares matrix: full	Secondary atom site location: difference Fourier map
*R*[*F*^2^ > 2σ(*F*^2^)] = 0.054	Hydrogen site location: inferred from neighbouring sites
*wR*(*F*^2^) = 0.139	H atoms treated by a mixture of independent and constrained refinement
*S* = 1.00	*w* = 1/[σ^2^(*F*_o_^2^) + (0.0472*P*)^2^] where *P* = (*F*_o_^2^ + 2*F*_c_^2^)/3
1937 reflections	(Δ/σ)_max_ < 0.001
227 parameters	Δρ_max_ = 0.18 e Å^−^^3^
2 restraints	Δρ_min_ = −0.17 e Å^−^^3^
0 constraints	

### Fractional atomic coordinates and isotropic or equivalent isotropic displacement parameters (Å^2^)

**Table:**

	*x*	*y*	*z*	*U*_iso_*/*U*_eq_	
O1	0.7382 (7)	0.4677 (2)	0.26965 (7)	0.0629 (8)	
O2	0.4317 (8)	0.3034 (3)	0.24222 (9)	0.0732 (10)	
H2	0.580 (6)	0.327 (5)	0.2350 (16)	0.110*	
O13	0.2881 (7)	0.5103 (3)	0.67472 (8)	0.0703 (9)	
O14	0.5820 (8)	0.6849 (3)	0.69302 (8)	0.0712 (10)	
H14	0.451 (8)	0.650 (4)	0.7037 (14)	0.107*	
C1	0.5599 (9)	0.4296 (3)	0.29987 (11)	0.0493 (11)	
C2	0.4047 (9)	0.3442 (3)	0.28494 (10)	0.0505 (11)	
C3	0.2159 (10)	0.2992 (3)	0.31135 (13)	0.0614 (12)	
H3A	0.1110	0.2419	0.3009	0.074*	
C4	0.1827 (10)	0.3406 (4)	0.35425 (11)	0.0619 (12)	
H4A	0.0536	0.3109	0.3723	0.074*	
C5	0.3379 (9)	0.4242 (4)	0.36995 (11)	0.0540 (11)	
C6	0.5250 (10)	0.4695 (3)	0.34251 (11)	0.0541 (11)	
H6A	0.6286	0.5275	0.3528	0.065*	
C7	0.3030 (9)	0.4706 (4)	0.41668 (11)	0.0676 (13)	
H7A	0.1446	0.4400	0.4292	0.081*	
H7B	0.2811	0.5506	0.4147	0.081*	
C8	0.5292 (10)	0.4456 (3)	0.44808 (11)	0.0516 (11)	
H8B	0.6859	0.4789	0.4352	0.062*	
C9	0.4850 (9)	0.5012 (3)	0.49355 (10)	0.0494 (10)	
H9A	0.3223	0.4712	0.5056	0.059*	
C10	0.7010 (10)	0.4725 (3)	0.52650 (10)	0.0656 (14)	
H10B	0.7041	0.3925	0.5306	0.079*	
H10C	0.8668	0.4941	0.5137	0.079*	
C11	0.6747 (9)	0.5273 (3)	0.57162 (11)	0.0556 (12)	
C12	0.4889 (9)	0.4893 (3)	0.60132 (11)	0.0549 (11)	
H12C	0.3845	0.4288	0.5941	0.066*	
C13	0.4594 (10)	0.5421 (3)	0.64204 (11)	0.0518 (11)	
C14	0.6091 (10)	0.6318 (3)	0.65279 (11)	0.0532 (11)	
C15	0.7953 (11)	0.6697 (3)	0.62395 (12)	0.0658 (12)	
H15B	0.8990	0.7304	0.6313	0.079*	
C16	0.8261 (11)	0.6157 (4)	0.58344 (12)	0.0634 (13)	
H16A	0.9535	0.6406	0.5639	0.076*	
C17	0.9001 (11)	0.5584 (4)	0.28192 (12)	0.0683 (13)	
H17B	1.0187	0.5746	0.2582	0.102*	
H17C	0.9968	0.5393	0.3081	0.102*	
H17D	0.7943	0.6225	0.2878	0.102*	
C18	0.5765 (13)	0.3217 (3)	0.45129 (11)	0.0801 (16)	
H18C	0.5897	0.2908	0.4220	0.120*	
H18D	0.4342	0.2873	0.4667	0.120*	
H18E	0.7355	0.3083	0.4672	0.120*	
C19	0.4509 (15)	0.6260 (3)	0.48960 (12)	0.0877 (18)	
H19D	0.4239	0.6573	0.5185	0.131*	
H19E	0.3030	0.6419	0.4712	0.131*	
H19F	0.6043	0.6580	0.4766	0.131*	
C20	0.1181 (10)	0.4196 (4)	0.66747 (13)	0.0668 (13)	
H20B	0.0149	0.4072	0.6936	0.100*	
H20C	0.2181	0.3541	0.6611	0.100*	
H20D	0.0058	0.4358	0.6429	0.100*	

### Atomic displacement parameters (Å^2^)

**Table:**

	*U*^11^	*U*^22^	*U*^33^	*U*^12^	*U*^13^	*U*^23^
O1	0.069 (2)	0.0660 (18)	0.0537 (14)	−0.0157 (19)	0.0004 (17)	−0.0018 (13)
O2	0.084 (3)	0.086 (2)	0.0494 (16)	−0.026 (2)	−0.0004 (17)	−0.0086 (14)
O13	0.078 (2)	0.082 (2)	0.0511 (14)	−0.022 (2)	0.0105 (16)	−0.0045 (14)
O14	0.090 (3)	0.070 (2)	0.0543 (16)	−0.020 (2)	0.0049 (18)	−0.0109 (14)
C1	0.049 (3)	0.054 (2)	0.045 (2)	−0.002 (2)	−0.008 (2)	0.0090 (18)
C2	0.055 (3)	0.053 (2)	0.044 (2)	0.001 (3)	−0.008 (2)	0.0010 (18)
C3	0.057 (3)	0.064 (3)	0.063 (2)	−0.011 (3)	−0.008 (2)	0.004 (2)
C4	0.049 (3)	0.080 (3)	0.056 (2)	−0.006 (3)	−0.007 (2)	0.008 (2)
C5	0.046 (3)	0.069 (3)	0.047 (2)	0.013 (3)	−0.005 (2)	−0.002 (2)
C6	0.049 (3)	0.061 (3)	0.052 (2)	0.002 (3)	−0.007 (2)	0.0010 (19)
C7	0.050 (3)	0.101 (3)	0.052 (2)	0.011 (3)	−0.004 (2)	−0.006 (2)
C8	0.047 (3)	0.063 (3)	0.0448 (19)	−0.004 (3)	0.008 (2)	0.0034 (18)
C9	0.045 (2)	0.058 (2)	0.0451 (19)	0.003 (3)	0.004 (2)	0.0060 (17)
C10	0.066 (3)	0.084 (3)	0.047 (2)	0.015 (3)	0.001 (2)	0.000 (2)
C11	0.055 (3)	0.067 (3)	0.045 (2)	0.010 (3)	−0.004 (2)	0.005 (2)
C12	0.059 (3)	0.055 (2)	0.051 (2)	0.001 (3)	−0.009 (2)	−0.0018 (19)
C13	0.052 (3)	0.059 (2)	0.044 (2)	0.002 (3)	−0.003 (2)	0.0064 (19)
C14	0.067 (3)	0.045 (2)	0.048 (2)	0.002 (3)	−0.002 (2)	0.0045 (19)
C15	0.075 (3)	0.063 (3)	0.060 (2)	−0.010 (3)	−0.001 (3)	0.009 (2)
C16	0.064 (3)	0.076 (3)	0.051 (2)	−0.007 (3)	0.006 (2)	0.013 (2)
C17	0.072 (4)	0.067 (3)	0.066 (2)	−0.016 (3)	−0.007 (3)	0.012 (2)
C18	0.116 (5)	0.066 (3)	0.058 (2)	0.016 (4)	0.002 (3)	−0.004 (2)
C19	0.122 (5)	0.068 (3)	0.073 (3)	0.025 (4)	−0.026 (3)	−0.005 (2)
C20	0.059 (3)	0.069 (3)	0.073 (3)	−0.014 (3)	−0.001 (3)	0.008 (2)

### Geometric parameters (Å, °)

**Table:**

O1—C1	1.371 (5)	C9—H9A	0.9800
O1—C17	1.420 (5)	C10—C11	1.518 (5)
O2—C2	1.386 (5)	C10—H10B	0.9700
O2—H2	0.84 (2)	C10—H10C	0.9700
O13—C13	1.376 (5)	C11—C16	1.364 (6)
O13—C20	1.414 (5)	C11—C12	1.386 (6)
O14—C14	1.378 (5)	C12—C13	1.391 (5)
O14—H14	0.86 (2)	C12—H12C	0.9300
C1—C2	1.375 (6)	C13—C14	1.363 (6)
C1—C6	1.384 (5)	C14—C15	1.371 (6)
C2—C3	1.367 (6)	C15—C16	1.393 (5)
C3—C4	1.396 (5)	C15—H15B	0.9300
C3—H3A	0.9300	C16—H16A	0.9300
C4—C5	1.368 (6)	C17—H17B	0.9600
C4—H4A	0.9300	C17—H17C	0.9600
C5—C6	1.380 (6)	C17—H17D	0.9600
C5—C7	1.526 (5)	C18—H18C	0.9600
C6—H6A	0.9300	C18—H18D	0.9600
C7—C8	1.529 (6)	C18—H18E	0.9600
C7—H7A	0.9700	C19—H19D	0.9600
C7—H7B	0.9700	C19—H19E	0.9600
C8—C18	1.512 (6)	C19—H19F	0.9600
C8—C9	1.542 (5)	C20—H20B	0.9600
C8—H8B	0.9800	C20—H20C	0.9600
C9—C19	1.516 (5)	C20—H20D	0.9600
C9—C10	1.529 (6)		
			
C1—O1—C17	118.3 (3)	C9—C10—H10C	108.6
C2—O2—H2	102 (4)	H10B—C10—H10C	107.5
C13—O13—C20	119.9 (3)	C16—C11—C12	118.7 (4)
C14—O14—H14	101 (3)	C16—C11—C10	121.4 (4)
O1—C1—C2	114.8 (3)	C12—C11—C10	119.8 (4)
O1—C1—C6	126.0 (4)	C11—C12—C13	119.6 (4)
C2—C1—C6	119.2 (4)	C11—C12—H12C	120.2
C3—C2—C1	121.1 (4)	C13—C12—H12C	120.2
C3—C2—O2	118.2 (4)	C14—C13—O13	114.2 (3)
C1—C2—O2	120.7 (4)	C14—C13—C12	120.7 (4)
C2—C3—C4	119.0 (4)	O13—C13—C12	125.1 (4)
C2—C3—H3A	120.5	C13—C14—C15	120.4 (4)
C4—C3—H3A	120.5	C13—C14—O14	121.3 (4)
C5—C4—C3	120.8 (4)	C15—C14—O14	118.3 (4)
C5—C4—H4A	119.6	C14—C15—C16	118.7 (4)
C3—C4—H4A	119.6	C14—C15—H15B	120.6
C4—C5—C6	119.2 (3)	C16—C15—H15B	120.6
C4—C5—C7	121.3 (4)	C11—C16—C15	121.8 (4)
C6—C5—C7	119.4 (4)	C11—C16—H16A	119.1
C5—C6—C1	120.7 (4)	C15—C16—H16A	119.1
C5—C6—H6A	119.7	O1—C17—H17B	109.5
C1—C6—H6A	119.7	O1—C17—H17C	109.5
C5—C7—C8	114.3 (4)	H17B—C17—H17C	109.5
C5—C7—H7A	108.7	O1—C17—H17D	109.5
C8—C7—H7A	108.7	H17B—C17—H17D	109.5
C5—C7—H7B	108.7	H17C—C17—H17D	109.5
C8—C7—H7B	108.7	C8—C18—H18C	109.5
H7A—C7—H7B	107.6	C8—C18—H18D	109.5
C18—C8—C7	110.8 (4)	H18C—C18—H18D	109.5
C18—C8—C9	113.2 (3)	C8—C18—H18E	109.5
C7—C8—C9	110.7 (4)	H18C—C18—H18E	109.5
C18—C8—H8B	107.3	H18D—C18—H18E	109.5
C7—C8—H8B	107.3	C9—C19—H19D	109.5
C9—C8—H8B	107.3	C9—C19—H19E	109.5
C19—C9—C10	111.0 (4)	H19D—C19—H19E	109.5
C19—C9—C8	112.1 (3)	C9—C19—H19F	109.5
C10—C9—C8	111.9 (3)	H19D—C19—H19F	109.5
C19—C9—H9A	107.2	H19E—C19—H19F	109.5
C10—C9—H9A	107.2	O13—C20—H20B	109.5
C8—C9—H9A	107.2	O13—C20—H20C	109.5
C11—C10—C9	114.9 (4)	H20B—C20—H20C	109.5
C11—C10—H10B	108.6	O13—C20—H20D	109.5
C9—C10—H10B	108.6	H20B—C20—H20D	109.5
C11—C10—H10C	108.6	H20C—C20—H20D	109.5
			
C17—O1—C1—C2	177.9 (4)	C18—C8—C9—C10	51.8 (6)
C17—O1—C1—C6	−1.7 (6)	C7—C8—C9—C10	176.9 (3)
O1—C1—C2—C3	−179.1 (4)	C19—C9—C10—C11	52.1 (5)
C6—C1—C2—C3	0.6 (6)	C8—C9—C10—C11	178.2 (4)
O1—C1—C2—O2	−0.6 (6)	C9—C10—C11—C16	−104.1 (5)
C6—C1—C2—O2	179.0 (4)	C9—C10—C11—C12	74.2 (5)
C1—C2—C3—C4	−0.5 (6)	C16—C11—C12—C13	0.5 (6)
O2—C2—C3—C4	−178.9 (4)	C10—C11—C12—C13	−177.8 (4)
C2—C3—C4—C5	−0.6 (6)	C20—O13—C13—C14	178.9 (4)
C3—C4—C5—C6	1.5 (6)	C20—O13—C13—C12	−2.3 (6)
C3—C4—C5—C7	179.7 (4)	C11—C12—C13—C14	0.8 (6)
C4—C5—C6—C1	−1.4 (6)	C11—C12—C13—O13	−177.9 (4)
C7—C5—C6—C1	−179.6 (4)	O13—C13—C14—C15	177.5 (4)
O1—C1—C6—C5	179.9 (4)	C12—C13—C14—C15	−1.5 (6)
C2—C1—C6—C5	0.3 (6)	O13—C13—C14—O14	−1.1 (6)
C4—C5—C7—C8	112.6 (5)	C12—C13—C14—O14	−180.0 (4)
C6—C5—C7—C8	−69.3 (5)	C13—C14—C15—C16	0.7 (7)
C5—C7—C8—C18	−56.9 (5)	O14—C14—C15—C16	179.2 (4)
C5—C7—C8—C9	176.7 (3)	C12—C11—C16—C15	−1.3 (6)
C18—C8—C9—C19	177.3 (5)	C10—C11—C16—C15	177.0 (4)
C7—C8—C9—C19	−57.6 (6)	C14—C15—C16—C11	0.7 (7)

### Hydrogen-bond geometry (Å, °)

**Table:**

*D*—H···*A*	*D*—H	H···*A*	*D*···*A*	*D*—H···*A*
O2—H2···O14^i^	0.84 (2)	2.15 (3)	2.908 (6)	149 (5)
O14—H14···O2^ii^	0.86 (2)	2.35 (4)	3.030 (5)	137 (5)
O2—H2···O1	0.84 (2)	2.14 (5)	2.658 (4)	119 (5)
O14—H14···O13	0.86 (2)	2.07 (4)	2.644 (5)	124 (4)

Symmetry codes: (i) −*x*+3/2, −*y*+1, *z*−1/2; (ii) −*x*+1/2, −*y*+1, *z*+1/2.
